# Mitochondrial function of immune cells in septic shock: A prospective observational cohort study

**DOI:** 10.1371/journal.pone.0178946

**Published:** 2017-06-07

**Authors:** Tobias M. Merz, Adriano J. Pereira, Roger Schürch, Joerg C. Schefold, Stephan M. Jakob, Jukka Takala, Siamak Djafarzadeh

**Affiliations:** 1 Department of Intensive Care Medicine, Bern University Hospital, University of Bern, CH-3010 Bern, Switzerland; 2 Division of Statistics, Clinical Trials Unit, Institute of Social and Preventive Medicine, University of Bern, Bern, Switzerland; University of the Pacific, UNITED STATES

## Abstract

**Background:**

Reduced cellular ATP synthesis due to impaired mitochondrial function of immune cells may be a factor influencing the immune response in septic shock. We investigate changes in mitochondrial function and bioenergetics of human monocytes and lymphocyte subsets.

**Methods:**

Thirty patients with septic shock were studied at ICU admission, after 24 and 48 hours, and after resolution of shock. Enzymatic activities of citrate synthase and mitochondrial complexes I, IV, and ATP synthase and ATP content of monocytes, T-cells and B-cells and pro-inflammatory (IL-1β and IL-6) and anti-inflammatory (IL-10) cytokine plasma concentrations were compared to samples from 20 healthy volunteers.

**Results:**

Large variations in mitochondrial enzymatic activities of immune cells of septic patients were detected. In monocytes, maximum levels of citrate synthase activity in sepsis were significantly lower when compared to controls (p = 0.021). Maximum relative enzymatic activity (ratio relative to citrate synthase activity) of complex I (p<0.001), complex IV (p = 0.017) and ATP synthase (p<0.001) were significantly higher. In T-cells, maximum levels of citrate synthase (p = 0.583) and relative complex IV (p = 0.602) activity did not differ between patients and controls, whereas levels of relative complex I (p = 0.006) and ATP synthase (p = 0.032) were significantly higher in septic patients. In B-cells of patients, maximum levels of citrate synthase activity (p = 0.004) and relative complex I (p<0.001) were significantly higher, and mean levels of relative complex IV (p = 0.042) lower than the control values, whereas relative ATP synthase activity did not differ (p = 1.0). No significant difference in cellular ATP content was detected in any cell line (p = 0.142–0.519). No significant correlations between specific cytokines and parameters of mitochondrial enzymatic activities or ATP content were observed.

**Conclusions:**

Significant changes of mitochondrial enzymatic activities occur in human peripheral blood immune cells in septic shock when compared to healthy controls. Assessed sub-types of immune cells showed differing patterns of regulation. Total ATP-content of human immune cells did not differ between patients in septic shock and healthy volunteers.

## Introduction

Mitochondria are key players in cellular energy metabolism by generation of cellular adenosine-5'-triphosphate (ATP) supply through oxidative phosphorylation. In sepsis, biochemical and ultrastructural abnormalities of mitochondria have been recognized in liver [1], kidney [2, 3], skeletal and heart muscle tissue [4], and blood cells [5, 6]. Oxidative phosphorylation and ATP generation are affected by depleted levels of reduced glutathione and by an increased generation of reactive oxygen species and reactive nitrogen species [4]. Additionally, impairment of the mitochondrial electron transport chain due to uncoupling of the oxidative phosphorylation as a result of uncoupling proteins [7], or the opening of the permeability transition pores [8], have been described in animal models of sepsis. This acquired intrinsic derangement in cellular energy metabolism impairs the activities of the mitochondrial electron transport chain enzyme complexes and ATP biosynthesis and may contribute to organ dysfunction in sepsis [9, 10].

Immune cells need energy in the form of ATP to sustain housekeeping functions including maintenance of ionic integrity, volume regulation and cell growth [11]. Additional specific immune processes, which largely depend on ATP as energy supply, include cellular migration and phagocytosis [12, 13], antigen processing and presentation [14], and effector functions such as synthesis of antibodies and cytotoxicity, as well as regulatory functions [15–17]. Septic shock induces an increase in baseline oxygen consumption in peripheral blood mononuclear cells (PBMC) [5]. However, a reduction in adenosine diphosphate (ADP)-induced maximal mitochondrial respiration and associated ATP synthesis occurs in sepsis, which is associated with sepsis severity and mortality [18]. Reduced maximal ATP synthesis due to impaired mitochondrial function of immune cells may therefore be a factor influencing the effectiveness of the immune response [11]. The underlying mechanisms leading to sepsis-associated impairment of mitochondrial function and reduction of ATP synthesis of immune cells are complex and still not completely understood. Different mechanisms have been proposed, including increased nitric oxide production and nitration of mitochondrial proteins [19], an increase in levels of anti-inflammatory cytokines [20], a reduction in the functional content of ATP synthase complex [18], and alterations in mitochondrial membrane potential [21]. Inhibition of ATP synthesis in immune cells may contribute to the often observed immune cellular anergy and impaired adaptive immune responses in patients with severe sepsis and septic shock [22–24] and to the down-regulation of immune-cell activity associated with prolonged sepsis and adverse outcomes [5, 18, 25, 26].

Previous studies have examined samples containing peripheral PBMC—monocytes and lymphocytes—without further separation into specific immune cell subpopulations [5, 18, 21, 27]. Therefore, data on mitochondrial function of specific human immune cell subtypes in severe sepsis is limited and the potential correlation of mitochondrial energy requirements and production, as well as disease severity and outcome is unclear. The immune response in the context of severe sepsis consists of an interdependent, highly-complex system that involves different populations of immune cells, as well as pro and anti-inflammatory cytokines in a time-dependent process. Typically, the adaptive immune responses, phenotypic changes and cytokine expression profiles differ between the specific immune cell types [28].

The aim of this observational study was to investigate mitochondrial function and bioenergetics of monocytes, B cells and CD4+ T-cells in correlation to pro- and anti-inflammatory cytokines in patients with septic shock versus healthy controls. We hypothesized that subtypes of immune cells feature differing patterns of regulation of mitochondrial enzymatic activities and that impaired cellular energy production might occur in the context of septic shock.

## Methods and materials

### Study design

Prospective single centre cohort study.

### Setting

The study was performed in the Department of Intensive Care Medicine of the Bern University Hospital, a 960-bed tertiary care centre in Switzerland. The Department of Intensive Care Medicine (ICU) is the sole provider of intensive care for adults at the hospital, handling all types of medical, surgical and trauma patients.

### Patients and subjects

A total of 30 adult patients (age > 18years) admitted to the intensive care unit (ICU) due to the recent onset (<24h since start of symptoms) of septic shock [29] were included in the study. Due to the logistics of sample preparation, cell line isolation and analytics, recruitment was limited to patients admitted on Monday-Thursday. With these limitations, consecutive patients were recruited. Septic shock was defined as presence of proven or suspected infection, new onset of dysfunction of at least one organ, a systolic arterial pressure <90 mm Hg or mean arterial pressure <60mmHg (despite adequate volume resuscitation of >30 mL/kg of crystalloids); and the absence of other causes for hypotension [29]. Patients with any type of chronic infectious, inflammatory or autoimmune diseases, patients after hematopoietic or solid organ transplantations and patients receiving long-term treatment with steroids or other immunosuppressive agents were excluded from the study. Patients were treated according to standard recommendations [30]. Twenty healthy volunteers from different age groups (20 years to 65 years, 10 female and 10 male) served as controls. Subjects with any kind of comorbidities, chronic underlying conditions or subjects on any kind of regular drug regime were not included in the study.

### Data collection

Collected baseline characteristics included patient demographics, diagnosis, Acute Physiology and Chronic Health Evaluation (APACHE II) score and Sequential Organ Failure Assessment (SOFA) score at ICU admission. Daily collected follow-up data included SOFA score, acute physiology parameters of APACHE II, and routinely collected clinical and laboratory parameters for assessment of circulatory, lung, cerebral, hepatic and renal function. The ICU uses a Patient Data Management System (PDMS) to collect and register data for clinical use. These data include all hemodynamic, respiratory and other clinical parameters, as well as all information on drug use and blood tests. Data from the PDMS were exported into a study data base.

### Blood sampling for study purposes

Study specific blood samples (30ml) were collected at the time of ICU admission (T0), after 24h (T1), 48h (T2) and after resolution of shock defined as mean arterial blood pressure >60mmHg without use of inotropes and/or vasopressors and absence of signs of organ dysfunction for at least 4 hours (T3). In the control group, blood samples were collected twice with a 7-day interval. Blood samples were collected into tubes containing EDTA, centrifuged immediately at 600 x G for 10 min at 4°C and the plasma fractions were apportioned into aliquots frozen immediately at -80°C.

### Isolation of human immune (Monocytes, B and T cells)

Isolation of immune cells was performed using an antibody-antigen mediated immunomagnetic cell isolation procedure that isolates specific target cells directly from whole blood (Dynabeads® Human CD14 Monocytes, Dynabeads® Human CD19^+^ B cells and Dynabeads® Human CD4^+^ T cells) (Thermo Fisher Scientific, USA) according to the manufacturer’s instructions. Preparation of Dynabeads: for each cell type isolation (monocytes, B cells or T cells), 100 μl of the corresponding Dynabeads was transferred to an eppendorf tube and 1 ml of phosphate buffer saline (PBS) supplemented with 0.1% BSA, pH 7.4 were added to the tubes and mixed. Afterwards the tubes were placed in a magnet (DynaMag™ Magnet) (Thermo Fisher Scientific, Beverly, MA, USA) for 1 min and the supernatants were discarded. Then, the tubes were removed from the magnet and the washed Dynabeads were resuspended in 100 μl of phosphate buffer saline (PBS) supplemented with 0.1% BSA, pH 7.4. Preparation of blood samples: the whole blood (10 ml blood per each cell type) was diluted with 20 ml PBS (without Ca^2+^ or Mg^2+^) supplemented with 0.1% BSA and 2 mM EDTA, centrifuged at 600 x g for 10 min at 4°C and the plasma fractions (upper layer) were discarded and the pellets were resuspended to the original volumes (10 ml each) in PBS supplemented with 0.1% BSA, pH 7.4. Incubation of blood samples with dynabeads: washed Dynabeads specific for monocytes, B and T cells (100 μl each) were added to the prepared blood samples in PBS (10 ml each) and incubated for 20 min at 4°C with gentle tilting and rotation. Then the tubes were placed in the magnet for 2 min, the supernatants were discarded and bead bound cells were washed 2 times by resuspending in 1 ml of PBS supplemented with 0.1% BSA, pH 7.4 and 2 times by resuspending in 1 ml of phosphate buffer saline (PBS), pH 7.4, and separated using the magnet. The bead-bound cells were frozen immediately at -80°C. The Isolation of human immune cells protocol was completed in approximately 35 min, including 10 min incubation of the blood and and 20 incubation phases with dynabeads.

### Cell lysis and determination of cellular protein content

The frozen bead-bound cells were thawed on ice and lysed in 320 μL of M-PER mammalian protein extraction reagent (Thermo Fisher Scientific, USA) and cellular protein content was measured using Pierce bicinchoninic acid (BCA) protein assay kit (Thermo Scientific, Rockford, IL, USA). To determine cellular ATP content 30 μL of cell lysate was deprotenized immediately and cellular ATP content was measured using the Molecular Probesʼ ATP Determination Kit (Invitrogen, Life Technologies, Zug, Switzerland). The rest of the cell lysates were used for the determination of mitochondrial complex I, IV, ATP synthase and citrate synthase enzyme activities.

### Determination of mitochondrial complex I, IV, ATP synthase and citrate synthase enzyme activities

The activities of cellular mitochondrial complexes I, and IV, ATP synthase and citrate synthase were assayed spectrophotometrically using Abcam’s microplate assay kits (Abcam Inc., Cambridge, England) according to the manufacturer's instructions. The assay kits are based on immunocapturing of mitochondrial enzyme complexes from cellular extracts by capture monoclonal antibodies which are pre-coated in the wells of premium Nunc MaxiSorp™ modular microplates. After the target enzyme has been captured in the well, specific substrates are added, and enzyme activity is analyzed by measuring the change in absorbance of either the substrate or the product of the reaction. Mitochondrial complex I enzyme activity was measured using Abcam’s complex I enzyme activity microplate assay Kit (ab109721) by following the oxidation of reduced nicotinamide adenine dinucleotide (NADH) to oxidized NAD^+^ and the simultaneous reduction of a dye which leads to increased absorbance at OD = 450 nm. Mitochondrial complex IV enzyme activity was measured colorimetrically using Abcam’s complex IV enzyme activity microplate assay Kit (ab109909) by following the oxidation of reduced cytochrome c by the absorbance change at 550 nm. Mitochondrial ATP synthase enzyme activity was measured colorimetrically using Abcam’s ATP synthase enzyme activity microplate assay Kit (ab109714). The assay is based on, the conversion of ATP to ADP by ATP synthase which is coupled to the oxidation reaction of NADH to NAD^+^ with a reduction in absorbance at 340 nm. Mitochondrial citrate synthase enzyme activity was measured colorimetrically using Abcam’s citrate synthase enzyme activity microplate assay Kit (ab119692) by recording color development of 5-thio-2-nitrobenzoic acid (TNB) which absorbs at OD = 412 nm, and is generated from 5,5'-dithio-bis-[2-nitrobenzoic acid]) (DTNB) present in the reaction of citrate synthesis. The activity rates of mitochondrial complex I, IV, ATP synthase and citrate synthase are expressed as the change in absorbance nm/minute/amount (mg) of sample loaded into the wells and for statistical analysis enzymatic activities of mitochondrial complex I, IV and ATP synthase are presented as their ratio to citrate synthase to account for changes in mitochondrial number and/or mass (relative enzymatic activity). The mitochondrial enzymatic activities of monocytes, T-cells and B-cells in one patient sample, activities of monocytes in two samples from volunteers and of T-cells and B-cells in three samples from volunteers were not detectable as activities fell below the detection limit of the analytical method due to the low number of isolated cells.

### Measurement of the cellular ATP content

To determine cellular ATP content first cell lysates were deprotenized using the Deproteinizing Sample Preparation Kit (BioVision, Milpitas, CA) and ATP content was measured using the Molecular Probesʼ ATP Determination Kit (Invitrogen, Life Technologies, Zug, Switzerland) according to the manufacturer’s instructions. The assay kit is based on firefly luciferase and the production of light caused by the reaction of ATP with added luciferase and D-luciferin. The emitted light is measured on a luminescent counter and is proportional to the ATP concentration inside the cell. The ATP content of monocytes and T-cells in one volunteer sample and of B-cells in seven volunteer samples were not detectable as ATP levels fell below the detection limit of the analytical method due to the low number of isolated cells.

### Measurement of soluble CD73

Soluble CD73 also known as 5-nucleotidase was measured in plasma of patients and volunteers spectrophotometrically using human 5-nucleotidase ELISA Kit (Wuhan Fine Biological Technology Co.,Ltd., Wuhan, Hubei, China) according to the manufacturer's instructions. The kit is based on sandwich enzyme-linked immune-sorbent assay technology. The standards, plasma samples and biotin conjugated detection antibody were added to the wells subsequently, and washed with wash buffer. Horseradish Peroxidase (HRP)-Streptavidin was added and unbound conjugates were washed away with wash buffer. 3,3',5,5'-Tetramethylbenzidine (TMB) substrates were used to visualize HRP enzymatic reaction. TMB was catalyzed by HRP to produce a blue color product that changed into yellow after adding acidic stop solution. The density of yellow is proportional to the 5-NT amount of sample captured in plate. The O.D. absorbance was read at 450nm in a microplate reader, and then the concentration of 5-NT was calculated.

### Measurements of pro- and anti-inflammatory cytokines levels

Pro-inflammatory (IL-1β and IL-6) and anti-inflammatory (IL-10) cytokine contents in plasma were measured by Enzyme-linked immunosorbent assay (ELISA) using the LEGEND MAX™ Human ELISA Kits (Biolegend, San Diego, CA, USA), according to the manufacturer's instructions. Each Kit is a Sandwich Enzyme-Linked Immunosorbent Assay (ELISA) with a 96-well strip plate that is pre-coated with a capture antibody (anti-human IL-10, IL-1β or IL-6 antibodies). Each kit is analytically validated with ready-to-use reagents and is specifically designed for the accurate quantitation of human IL-1β, IL-6 or TNF-α from cell culture supernatant, serum or plasma. Briefly, each well in each IL-1β, IL-6 or TNF-α 96-well ELISA plates is washed 4 times with 300 μL of ready-to-use wash buffer (supplied in the kit). Afterwards, 50 μL of diluted IL-10, IL-1β or IL-6 standards or human plasma samples (in duplicates) are added into the wells and incubated at room temperature for 2 hours while shaking at 200 rpm. Then, the contents of the wells are discarded and the wells are washed 4 times with 300 μL of wash buffer and 100 μL of human IL-1β, IL-6 or TNF-α detection antibody solution is added into each well and the plates are incubated at room temperature for 1 hour while shaking. Then, the wells are washed 4 times with 300 μL of wash buffer and 100 μL of avidin-horseradish peroxidase (HRP) A solution is added into each well and incubated at room temperature for 30 minutes while shaking. Finally, the wells are washed again 4 times with 300 μL of wash buffer and 100 μL of ready-to-use substrate solution buffer (supplied in the kit) is added into each well and incubated for 20 min at room temperature in the dark. Afterwards the reaction is stopped by adding 100 μL of stop solution (supplied in the kit) to each well and the absorbance is read at 450 nm within 30 minutes. The cytokine concentrations were calculated with computer-based curve-fitting software using a 4-parameter logistics curve-fitting algorithm. If a plasma sample’s absorbance value was outside the linear portion of the standard curve, the sample was re-analyzed at a higher (or lower) dilution as appropriate.

### Study size and definition of measurement intervals

Data on scale and variance of changes of mitochondrial enzymatic activities in human immune cells during sepsis are not reported in the literature, therefore the study size could not be based on a power analysis. The study size was determined based on the expected number of patients who could be included in our institution within a recruitment period of 18 months. Time intervals between measurements were defined based on the expected average duration of circulatory failure of survivors of septic shock, serial sampling was applied to investigate temporal patterns of mitochondrial enzymatic activities, ATP content and cytokine levels during evolution of septic shock and to identify the highest levels.

### Statistical analysis

Characteristics and baseline laboratory data of patients in septic shock are reported stratified by survival. We show means ± SD or medians (IQR), or percentages, as appropriate. We compared non-survivors and survivors using t-tests, Wilcoxon rank sum tests (W) for unpaired data, or χ^2^-test. Differences in mitochondrial enzymatic activities, ATP content, soluble CD73 levels, and cytokine levels between patients and volunteers were assessed using Wilcoxon rank sum tests. The maximal values for mitochondrial enzymatic activity values, ATP content, soluble CD73 levels and cytokine levels occurring at any time point T0 to T3 was used for each measurement and patient. For comparison purposes, the mean values of the two samples collected with a 7 days interval were used for measurements in healthy volunteers. Linear mixed-effect models were used to assess temporal changes of mitochondrial activities and ATP content and differences between survivors and non-survivors of septic shock; results are reported as F value and significance p. Potential temporal curve changes based on the four measurement time points were assessed by fitting growth curves up to a third order polynominal as a covariate and survival as fixed effect. Spearman rank correlations among mitochondrial enzymatic activities and single cytokine levels, as well as ratios of inflammatory and anti-inflammatory cytokines (IL-6/IL-10) in septic patients were explored plotting pairwise comparisons and calculating Spearman rank correlations at time T0. We used the method of Benjamini and Hochberg (1995) to correct for false discovery rates [31]. For all analyses, missing data due to patients’ death or due to difficulties in sample processing was not imputed as the applied statistical methods estimate parameters directly using all the information contained in incomplete data sets. The strength of the correlations and whether the association was significant was visualized using *corrgram*. We used SPSS Version 21 and R 3.1.1 for all statistical analyses, and for creating the figures.

### Ethical approval and patient consent

The institutional review board of the Canton of Bern reviewed and approved the study (KEK 015/10). Patients were included into the study at the time of ICU admission. Written deferred proxy consent was obtained as soon as possible after contacting the next of kin followed by written informed consent of the patient. Written informed consent was obtained from all subjects of the control group before study inclusion.

### Trial registration

ClinicalTrials.gov Identifier: NCT01600989.

## Results

### Patients

A total of 30 patients in septic shock were included in the study. Of these, 22 survived to hospital discharge, whereas 8 died after a length of stay of 31.2 [7.8, 67.0] hours. Six patients died before T2 sampling and one patient before T3 sampling. Non-survivors had significantly more comorbidities and higher APACHE II scores, whereas occurrence of failure of single organs and SOFA scores were comparable. The baseline characteristics of the included patients are shown in Table 1. An additional file in the electronic supplement reports all clinical data in more detail (S1 Table).

**Table 1:** Characteristics of surviving and non-surviving septic patients.

	non-survivors	survivors	p
Age	65.5±8.5	63.5±17.2	0.684
Gender (males)	6 (75.0)	12 (54.5)	0.555
Acute kidney injury (present)	5 (62.5)	12 (54.5)	1.000
Abrupt mental change (present)	1 (12.5)	2 (9.1)	1.000
Septic shock (present)	8 (100.0)	22 (100.0)	-
Source of infection			0.847
• pulmonary	4 (50.0)	8 (36.4)	
• gastrointestinal	3 (37.5)	5 (22.7)	
• soft tissue	1 (12.5)	4 (18.2)	
• cardio-vascular	0 (0.0)	2 (9.1)	
• central nervous system	0 (0.0)	1 (4.5)	
• genito-urinary	0 (0.0)	1 (4.5)	
• other	0 (0.0)	1 (4.5)	
Time first documented sign of sepsis to ICU admission (h)	5.5 [3.8, 8.9]	13.7 [4.0, 28.2]	0.152
Time organ failure to start antibiotics (h)	1.7 [1.3, 2.7]	2.1 [1.0, 5.0]	0.656
Time organ failure to start surgery (h)	6.6 [3.1, 11.0]	2.8 [-0.4, 5.0]	0.170
Surgery required (yes)	5 (62.5)	12 (54.5)	1.000
Charlson co-morbidity index	2.0 [1.0, 2.2]	0.5 [0.0, 1.0]	0.010
APACHE II at ICU admission	31.0 [27.0, 36.2]	21.5 [18.2, 26.5]	0.013
SOFA at ICU admission	10.0 [8.0, 10.8]	10.0 [8.0, 11.0]	0.830
ALI or ARDS at ICU admission (yes)	7 (87.5)	17 (77.3)	0.918
White blood cell count at ICU admission (G/L, mean ± standard deviation)	14.2 ± 8.1	13.8 ± 13.8	0.912
C reactive protein at ICU admission (mg/L, mean ± standard deviation)	179 ± 129	199 ± 103	0.664

### Monocytes

**Fig 1:**
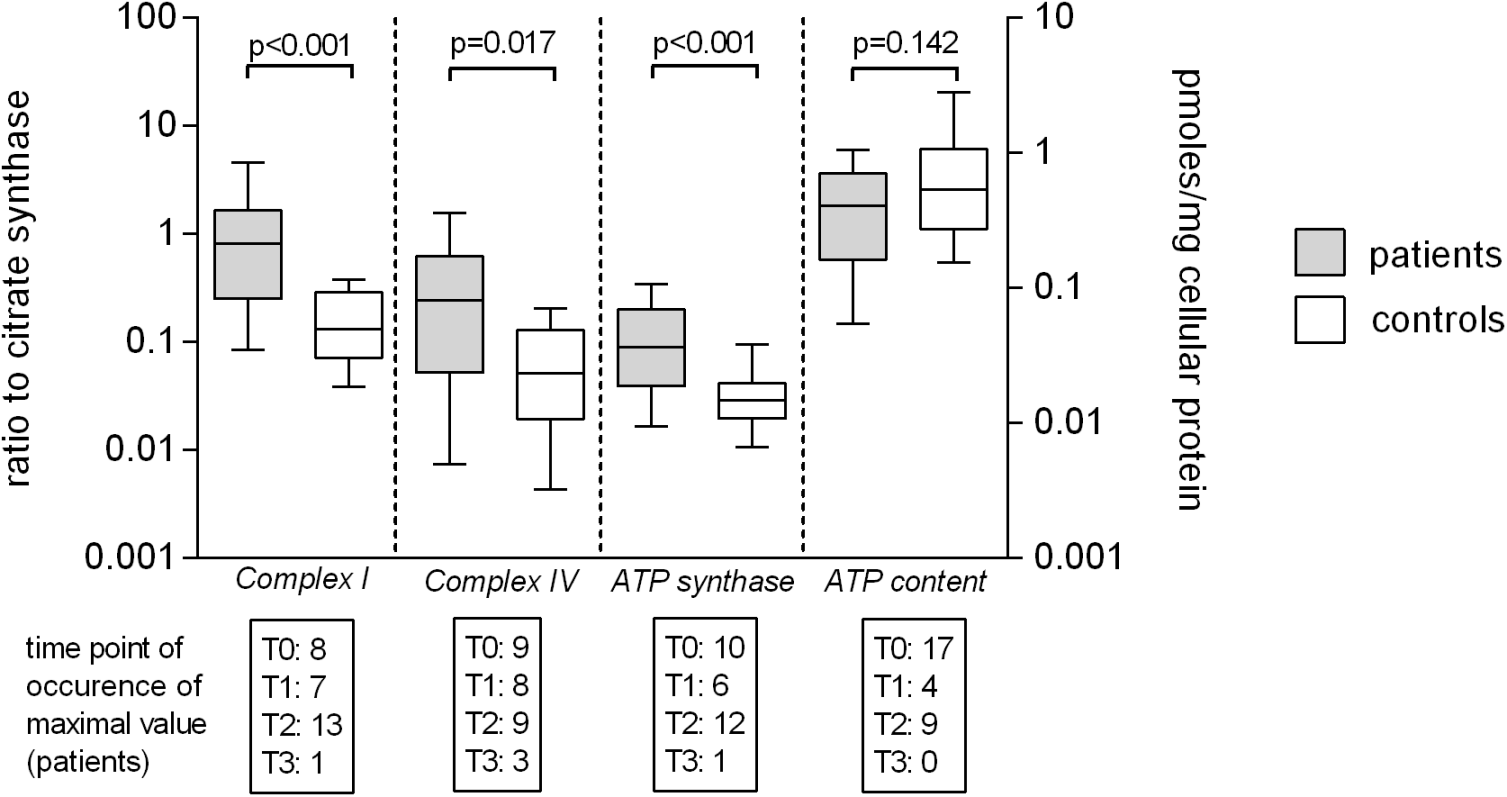
Mitochondrial enzymatic activity and cellular ATP content of monocytes of patients and volunteers. Maximum enzymatic activities during evolution of septic shock of complex I, complex IV and ATP synthase and maximum levels of APT content of monocytes of patients versus control values. Maximum enzymatic activities of are presented as ratios to citrate synthase (complex enzyme activity/citrate synthase) to account for mitochondrial matrix density (left Y-axis) and were available for 29 patients and 18 controls. Maximum cellular ATP content is expressed as pmol/mg cellular protein (right Y-axis) and was available for 30 patients and 19 controls. Numbers in boxes below X-axis indicate in how many patients a maximum level was measured at which time point (T0 to T3) stratified by single enzymatic activity and ATP content. Data is given as median and interquartile range (box), error bars indicate the 95% CI of values.

Maximum levels of citrate synthase activity of patients in septic shock (median 114 mOD/min/mg cellular protein, IQR 60–176) as a marker enzyme for the mitochondrial matrix representing mass and/or number of mitochondria were significantly lower than in healthy volunteers (median 180 mOD/min/mg cellular protein, IQR 134–224) (p = 0.021). Maximum relative enzymatic activities (complex enzyme activity/citrate synthase) of complex I (patients 0.810, IQR 0.250–1.655; controls 0.130, IQR 0.071–0.286), complex IV (patients 0.242, IQR 0.052–0.619; controls 0.051, IQR 0.019–0.129) and ATP synthase (patients 0.089, IQR 0.039–0.198; controls 0.029, IQR 0.020–0.042) were significantly higher in septic patients versus controls. Cellular ATP content (patients 0.531 pmoles/mg cellular protein, IQR 0.273–1.065; controls 0.401 pmoles/mg cellular protein IQR 0.162–0.707) did not differ between patients and control values (Fig 1). We did not detect significant linear, quadratic of cubic trends to fit the pattern of changes of enzymatic activities or ATP content over time. Survivors and non-survivors of septic shock did not differ in relative enzymatic activities of complex I, complex IV or ATP synthase, enzymatic activity of citrate synthase or ATP content (Table 2).

**Table 2:** Temporal changes of mitochondrial enzymatic activities of monocytes and stratification by patient survival.

	relative enzymatic activities of						citrate synthase activity		ATP content	
	complex I		complex IV		ATP synthase					
	F	p	F	p	F	p	F	p	F	p
survival	0.477	0.495	0.090	0.766	0.007	0.934	0.371	0.548	0.234	0.632
Time	0.000	0.993	1.121	0.298	0.581	0.449	0.027	0.870	3.133	0.084
Time^2^	0.014	0.908	0.921	0.345	0.329	0.569	0.077	0.782	2.424	0.127
Time^3^	0.040	0.842	0.844	0.365	0.190	0.665	0.099	0.755	1.956	0.169

F, parameter estimates; p, significance. Time denotes linear time trend, Time^2^ denotes quadratic trend, Time^3^ denotes cubic trend

### T-cells

**Fig 2:**
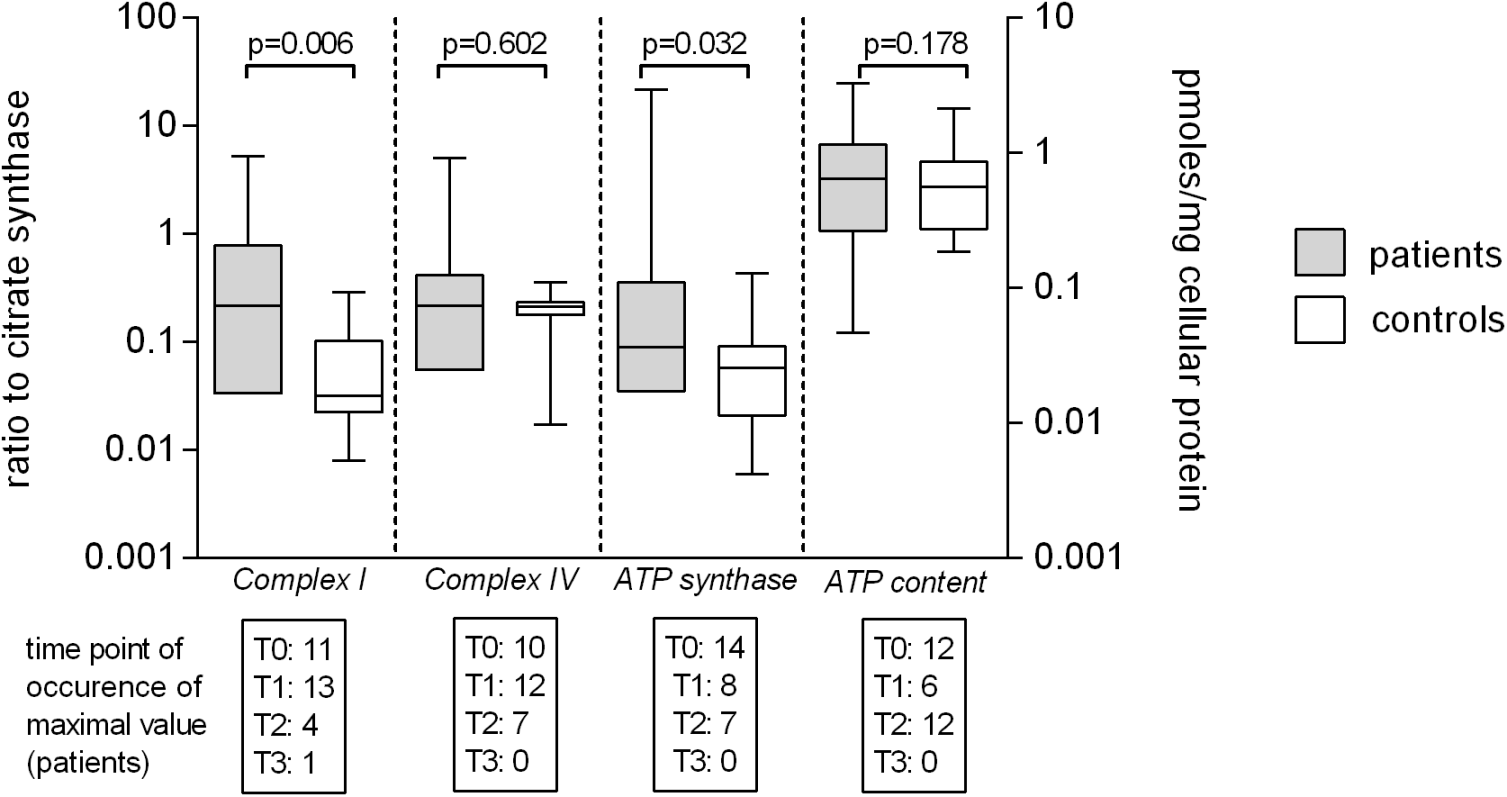
Mitochondrial enzymatic activity and cellular ATP content of T-cells of patients and volunteers. Maximum enzymatic activities during evolution of septic shock of complex I, complex IV and ATP synthase and maximum levels of APT content of T-cells of patients versus control values. Maximum enzymatic activities of are presented as ratios to citrate synthase (complex enzyme activity/citrate synthase) to account for mitochondrial matrix density (left Y-axis) and were available for 29 patients and 17 controls. Maximum cellular ATP content is expressed as pmol/mg cellular protein (right Y-axis) and was available for 30 patients and 19 controls. Numbers in boxes below X-axis indicate in how many patients a maximum level was measured at which time point (T0 to T3) stratified by single enzymatic activity and ATP content. Data is given as median and interquartile range (box), error bars indicate the 95% CI of values.

Maximum levels of citrate synthase activity did not differ between patients and control values (255mOD/min/mg cellular protein IQR 88–395 and 241mOD/min/mg cellular protein IQR 167–375; p = 0.583). We observed significantly higher maximum levels of relative enzymatic activities (complex enzyme activity/citrate synthase) of complex I (patients 0.214, IQR 0.033–0.784; controls 0.031, IQR 0.022–0.102) and ATP synthase (patients 0.088, IQR 0.035–0.365; controls 0.058, IQR 0.021–0.090) in patients than in controls, whereas complex IV (patients 0.214, IQR 0.055–0.416; controls 0.213, IQR 0.176–0.232) did not differ. Cellular ATP content (patients 3.193 pmoles/mg cellular protein, IQR 1.068–6.728; controls 2.691 pmoles/mg cellular protein, IQR 1.107–4.637) did not differ between patients and control values (Fig 2). We did not detect that linear, quadratic of cubic trends described the pattern of data over time. Linear mixed modelling detected an overall significant difference in complex I and ATP synthase enzymatic activity at different time points in survivors and non-survivors of septic shock (Table 3). This was mainly caused by differences in relative enzymatic activity of complex I (survivors 0.254, IQR 0.103–0.696; non-survivors 0.018, IQR 0.001–1.070), ATP synthase (survivors 0.086, IQR 0.044–0.451; non-survivors 0.072, IQR 0.014–27.550) at the time point T0.

**Table 3:** Temporal changes of mitochondrial enzymatic activities of T-cells and stratification by patient survival.

	relative enzymatic activities of						citrate synthase activity		ATP content	
	complex I		complex IV		ATP synthase					
	F	p	F	p	F	p	F	p	F	p
survival	8.051	0.008	2.980	0.095	14.727	0.001	3.311	0.075	0.325	0.541
Time	0.063	0.803	0.198	0.659	0.691	0.417	0.000	0.984	3.031	0.089
Time^2^	0.040	0.842	0.097	0.756	0.810	0.379	0.008	0.927	3.094	0.086
Time^3^	0.006	0.941	0.027	0.870	0.836	0.370	0.054	0.817	3.053	0.087

F, parameter estimates; p, significance. Time denotes linear time trend, Time^2^ denotes quadratic trend, Time^3^ denotes cubic trend

### B-cells

**Fig 3:**
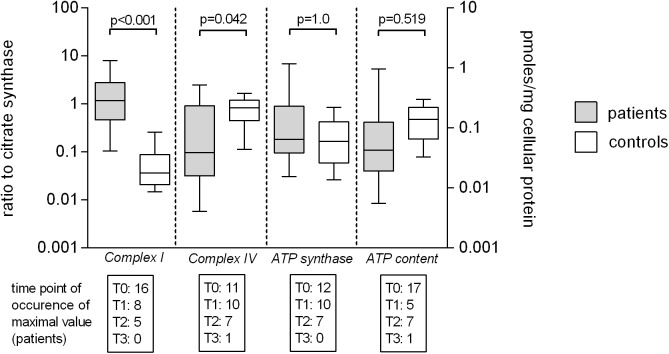
Mitochondrial enzymatic activity and cellular ATP content of B-cells of patients and volunteers. Maximum enzymatic activities during evolution of septic shock of complex I, complex IV and ATP synthase and maximum levels of APT content of B-cells of patients versus control values. Maximum enzymatic activities of are presented as ratios to citrate synthase (complex enzyme activity/citrate synthase) to account for mitochondrial matrix density (left Y-axis) and were available for 29 patients and 17 controls. Maximum cellular ATP content is expressed as pmol/mg cellular protein (right Y-axis) and was available for 30 patients and 13 controls. Numbers in boxes below X-axis indicate in how many patients a maximum level was measured at which time point (T0 to T3) stratified by single enzymatic activity and ATP content. Data is given as median and interquartile range (box), error bars indicate the 95% CI of values.

Maximum levels of citrate synthase activity of patients in septic shock (median 280mOD/min/mg cellular protein IQR 106–710) were significantly higher than in controls (median and 71mOD/min/mg cellular protein IQR 27–100) (p = 0.004). Maximum relative activities (complex enzyme activity/citrate synthase) of complex I (patients 1.164, IQR 0.460–2.783; controls 0.036, IQR 0.020–0.088) were significantly higher and of complex IV (patients 0.096, IQR 0.031–0.917; controls 0.823, IQR 0.443–1.191) were lower than in healthy controls, whereas ATP synthase activities (patients 0.181, IQR 0.095–0.894; controls 0.166, IQR 0.059–0.424) and ATP content (patients 0.097 pmoles/mg cellular protein, IQR 0.028–0.317; controls pmoles/mg cellular protein 0.189, IQR 0.001–0.564) did not differ (Fig 3). We did not detect that linear, quadratic of cubic trends described the pattern of data over time. Survivors and non-survivors of septic shock did not differ in relative enzymatic activities of complex I, complex IV or ATP synthase, enzymatic activity of citrate synthase or ATP content (Table 4).

**Table 4:** Temporal changes of mitochondrial enzymatic activities of B-cells and stratification by patient survival.

	relative enzymatic activities of						citrate synthase activity		ATP content	
	complex I		complex IV		ATP synthase					
	F	p	F	p	F	p	F	p	F	p
survival	1.427	0.237	0.277	0.600	0.169	0.684	2.176	0.144	0.043	0.837
Time	1.589	0.212	0.115	0.735	0.164	0.688	0.408	0.525	1.370	0.252
Time^2^	1.428	0.237	0.005	0.946	0.245	0.623	0.295	0.589	1.258	0.271
Time^3^	1.319	0.255	0.003	0.956	0.242	0.625	0.228	0.634	1.277	0.266

F, parameter estimates; p, significance. Time denotes linear time trend, Time^2^ denotes quadratic trend, Time^3^ denotes cubic trend

### Cytokine levels

Levels of IL-1β, IL-10 and IL-6 were significantly higher in septic patients in comparison to controls (Table 5).

**Table 5:** Comparison of mean and maximum cytokine activity of patients and values of healthy volunteers.

	maximum				mean			
	Volunteers	Patients	Comparison		Volunteers	Patients	comparison	
	(n = 20)	(n = 30)			(n = 20)	(n = 30)		
cytokine	median [IQR]	median [IQR]	W	p	median [IQR]	median [IQR]	W	p
IL-10 (pg/ml)	4.6 [3.1, 5.7]	143.6 [45.4, 391.3]	16	<0.001	3.8 [3.0, 4.6]	79.2 [22.5, 126.5]	30	<0.001
IL-1beta (pg/ml)	0.3 [0.3, 0.3]	1.6 [0.9, 4.8]	2	<0.001	0.3 [0.3, 0.3]	1.2 [0.8, 4.4]	2	<0.001
IL-6 (pg/ml)	2.4 [2.0, 2.7]	949.8 [253.9, 1392.6]	0	<0.001	2.2 [1.9, 2.5]	488.8 [171.7, 970.8]	0	<0.001

### Association among mitochondrial enzymatic and cytokine activities

**Fig 4:**
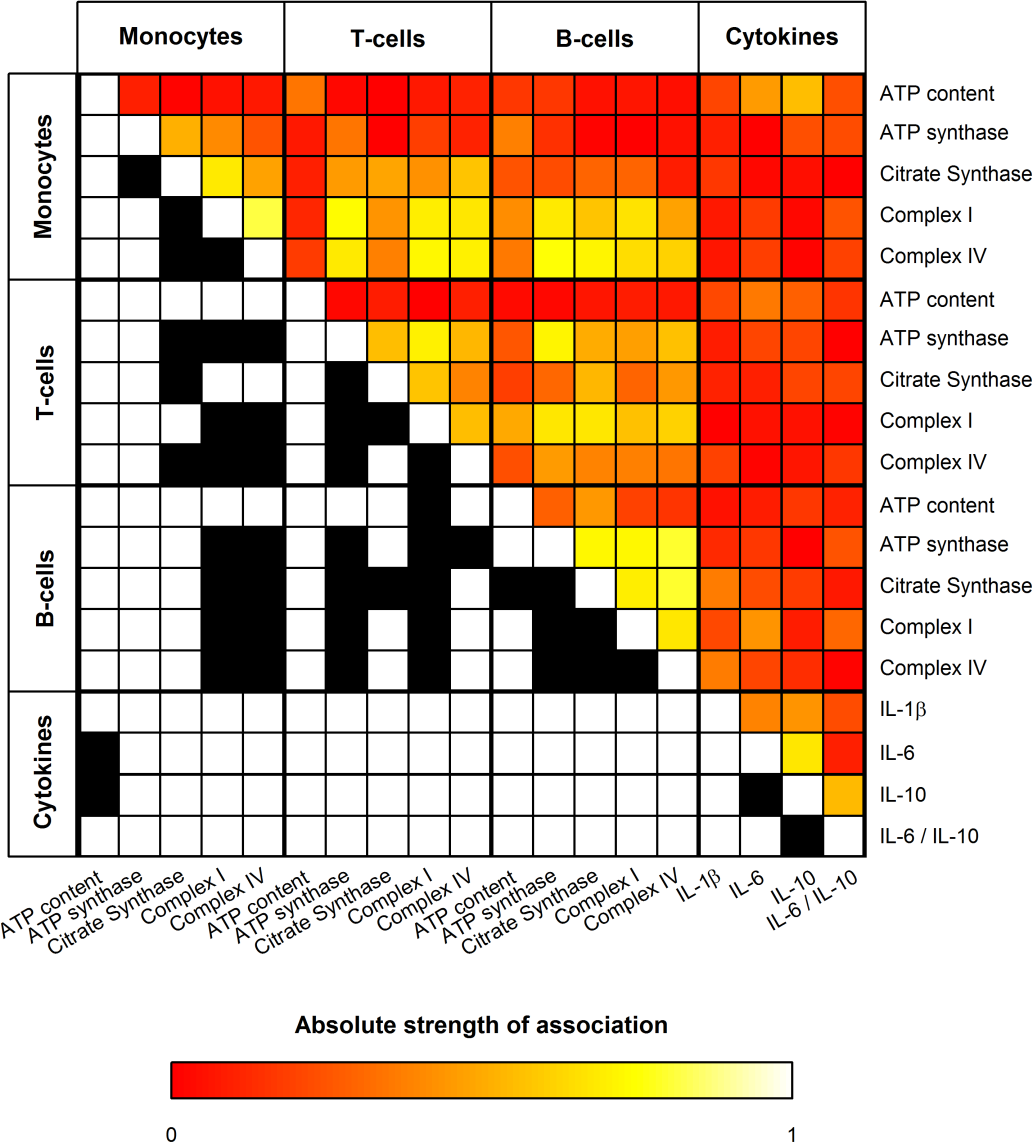
Strength of association among mitochondrial enzymatic activity and cellular ATP content, as well cytokine activity. Correlogram showing the strength of association among relative mitochondrial enzymatic activity (complex enzyme activity/citrate synthase)and cellular ATP content, cytokine activity, and the ratio of pro- and anti-inflammatory markers IL-6 / IL-10. Colors from red over orange to yellow indicate the strength of association from low to high. The black squares in the matrix denote correlations that were significant after accounting for false discovery rat.

Fig 4 shows that at T0 there are significant associations among immune cell mitochondrial enzymatic activities in septic patients. After considering multiple comparisons, no obviously relevant associations between immune cell activities and pro and anti-inflammation markers were detected.

### Levels of soluble CD73

The maximal level of soluble CD73 was significantly higher in patients compared to healthy controls (median 2.96 ng/ml IQR 1.21–5.20 vs 0.44 ng/ml IQR 0.28–1.10, p<0.0001).

### Availability of data

All study specific measurements of mitochondrial enzymatic activities, ATP content and cytokine activities are reported in detail in the electronic supplement (see Supporting Information S1 Table).

## Discussion

We found significant changes of mitochondrial enzymatic activities in human peripheral blood immune cells in septic shock when compared to healthy controls. Assessed sub-types of immune cells of septic patients showed differing patterns of regulation of mitochondrial enzymatic activities. Importantly, total ATP-content of human immune cells did not differ between patients in septic shock and healthy volunteers.

Our study has a number of limitations. The study included consecutive patients admitted to the ICU with the diagnosis of septic shock. As in most studies in this particular population of critically ill patients, the studied population was heterogeneous in terms of severity of shock, extent of organ failure, source of infection as well as age and pre-existing comorbidities. It is conceivable that these factors result in varying effects on mitochondrial function of immune cells and on levels of cytokines [32]. However, current theories about the onset and progression of sepsis focus on a uniform clinical syndrome that results from a dysregulated inflammatory response to any infection that is non-resolving and deleterious [33]. The extent of changes in mitochondrial function might be influenced by the time interval between onset of infection and sepsis and the time of the study assessment [24]. To minimize this effect we included only patients with acute onset (less than 24h after start of first symptoms) of sepsis and used serial blood sampling. Despite these efforts, we cannot exclude the possibility that measurements occurred at different time points during the evolution of septic shock and that this partially explains that we were not able to detect temporal patterns in changes of enzymatic activities or ATP content. Furthermore, a substantial number of patients died before completion of the study period and therefore blood samples were not obtainable for all pre-specified time points. This led to some missing data, which is an inevitability in septic shock studies. In the present study and frequently in many other studies mitochondrial respiratory chain enzyme activities are expressed relative to the citrate synthase enzyme activity [34–36]. Citrate synthase is a marker for the mitochondrial matrix. Citrate synthase activity and content depend on the number of mitochondria. The normalization of mitochondrial respiratory chain enzyme activities relative to citrate synthase will exclude any effect of the mitochondrial number and content on the activities of mitochondrial electron transport chain complexes. Moderate changes in activity of the single complexes may have little effect and lead to overestimation of effects on overall mitochondrial function. Secondly, mitochondrial function may also depend on the integrity of other processes not assessed by our methodology [37]. Measuring basal respiration and maximum respiration rate using high-resolution respirometry–as reported in previous studies in PBMCs [5, 18, 27, 38]—may have provided a better way to gain insight into cellular energetic function[39, 40]. However, polarographic measurement of oxygen consumption would have required excessively large sample volumes of blood to enable simultaneous measurements in specific immune cell sub-types. Cellular oxygen measurement technique provides information on mitochondrial integrity, maximal respiratory capacities of the electron transport chain complex and oxidative phosphorylation capacity. Spectrophotometric based enzymatic assays provide information on maximal mitochondrial electron transport chain complexes catalytic activities, require small amount of cells, and can be assessed using frozen cells. Lastly, the control group was recruited from healthy volunteers without any underlying chronic conditions or comorbidities to establish normal values of mitochondrial enzymatic activities in humans. We did abstain from including volunteers matched to patients regarding underlying conditions or to use patients with a similar level of organ failure do to conditions other than sepsis (such as trauma or post major surgery) as comparators. The aim of the study was to compare changes in mitochondrial enzymatic activities in septic shock versus health. Based on our data we can therefore not exclude the possibility that other factors other than sepsis–such as chronic conditions or presence of organ failure–caused the observed differences between patients and controls. However, including volunteers with matched chronic conditions or patients with matched organ failure severity of non-septic origin would have introduced significantly more heterogeneity within the control group, making comparisons to patients less precise and potentially impossible. Reference values were established in a limited number of subjects in the control group. Still, the non-parametric comparison of healthy volunteers and patients using Wilcoxon rank sum tests provide qualitatively equivalent results.

We are not aware of any previous studies assessing mitochondrial function in subtypes of immune cells in the context of severe sepsis or septic shock in humans. A few previous studies in septic patients reported results of high-resolution respirometry of PBMC isolated by density gradient centrifugation [5, 18, 27, 38, 41], which separates mononuclear from polynuclear cells and platelets. Belikova et al. found an increase in oxygen consumption of PBMCs in a cohort of 18 septic shock patients compared to healthy controls [5]. Stimulation of PBMCs by ADP produced a significantly lower increase in maximum oxygen consumption in cells of septic patients compared to cells from healthy controls, which indicates a decrease in bioenergetic reserve of septic cells. In contrast, Sjövall et al. found an increase in basal and maximum respiration in PBMCs of patients in septic shock compared to healthy controls. This might indicate an increased efficacy of mitochondria in the context of severe sepsis. Garrabou et al. reported that enzymatic mitochondrial complexes I, III, and IV and oxygen consumption were significantly inhibited in PBMCs in a cohort of 19 septic patients not in shock [41]. In a paediatric cohort of septic shock patients, Weiss et al. did not find a significant difference in basal or adenosine triphosphate (ATP)–linked oxygen consumption [38]. In contrast to the aforementioned reports, the antibody-antigen mediated immunomagnetic cell isolation used in our study allowed us to assess mitochondrial function of monocytes and T- and B-cells separately.

Monocytes are key antigen-presenting cells and represent a pivotal early defence system against pathogens in the context of sepsis [42]. This subpopulation of PBMC greatly expands in sepsis and the increase in absolute number is stimulated by pro-inflammatory cytokines [43]. Intact mitochondrial respiratory capacity and ATP production is a critical determinant of cell survival of immune cells [44]. If the mitochondrial respiratory capacity does not meet the stress-induced increase in metabolic demand, a depletion of cellular energy supply occurs, and this subsequently leads to cellular dysfunction which may present clinically as immune dysregulation or suppression [38]. Our findings show that higher enzyme activity of the mitochondrial electron transport system (ETS) of monocytes is present early in sepsis, potentially indicating increased cellular ATP production. In monocytes, we detected a significantly lower mitochondrial matrix density, which indicates a loss in in number and/or size of mitochondria. However, ATP content remains stable, suggesting an increase in ATP production which is counterbalanced by a sepsis-induced increased metabolic demand and therefore increased ATP consumption and/or release of ATP to the extracellular space. Immune cells respond to stimulation of specific pathogen recognizing surface receptors with the release of cellular ATP [45] leading to autocrine stimulation of purinergic receptors which regulate immune functions such as chemotaxis, antigen recognition [46], release of inflammatory cytokines [47] and elimination of bacteria [48] in autocrine and paracrine fashions. CD73 is a nucleotide metabolizing enzyme anchored to the plasma membrane of immune cells which catalyzes the stepwise hydrolysis of extracellular ATP to adenosine. Increased extracellular ATP stimulates monocytes to release of proinflammatory cytokines, such as IL-1beta and IL-6. Additionally, expression and function of CD 73 is upregulated leading to a more rapid conversion of ATP into adenosine [49] which in turn can augment release of anti-inflammatory cytokines (Il-10) [50]. The increased levels of pro- and anti-inflammatory cytokines and of soluble CD73 in septic patients compared to healthy controls might therefore indirectly indicate higher levels of extracellular ATP [49].

We did not document significant differences in mitochondrial enzymatic activities between survivors and non-survivors in immune cells of our patients in septic shock. This does not exclude that bioenergetic failure of immune cells contributes to an adverse outcome. Considering the heterogeneity of the study population and the high variability of measurement results, our study may have been insufficiently powered to detect differences determined by the severity of sepsis. Additionally it is conceivable that factors not influencing bioenergetics of immune cells and therefore not represented by our measurements could have had a major impact on the patient’s survival. In contrast to monocytes, we did not observe consistent up-regulation of mitochondrial enzymatic activities in B-cells and T-cells in patients with septic shock. These cells represent the main effector cells of the humoral and the cell-mediated immune response of the adaptive immune system. The acquired immune response is triggered by foreign cognate antigen presentation by antigen presenting cell (e.g. monocytes or dendritic cells) and generates a tailored reaction to eliminate specific pathogens. The acquired immune response might not lead to a simultaneous and uniform up-regulation of mitochondrial activities in B-cells and T-cells [51]. Our results do not indicate a correlation between single cytokines or ratios of inflammatory and anti-inflammatory cytokines and mitochondrial enzymatic activities or ATP content in any of the examined cell lines.

In conclusion, we observed a marked up-regulation of mitochondrial enzymatic activity indicating increased ATP production in monocytes, but not in B or T-cells. ATP content remained stable in human peripheral blood immune cells and does not significantly differ over the course of septic shock when compared to healthy volunteers.

## Supporting information

S1 Table Clinical parameters and laboratory measurements tables. (XLSX)
